# Identification of neurodegeneration indicators and disease progression in metachromatic leukodystrophy using quantitative NMR‐based urinary metabolomics

**DOI:** 10.1002/jmd2.12273

**Published:** 2022-01-27

**Authors:** Lucia Laugwitz, Laimdota Zizmare, Vidiyaah Santhanakumaran, Claire Cannet, Judith Böhringer, Jürgen G. Okun, Manfred Spraul, Ingeborg Krägeloh‐Mann, Samuel Groeschel, Christoph Trautwein

**Affiliations:** ^1^ Department of Neuropediatrics, Developmental Neurology and Social Pediatrics University of Tuebingen Tuebingen Germany; ^2^ Bruker BioSpin GmbH Ettlingen Germany; ^3^ Dietmar‐Hopp Metabolic Center Children's Hospital Heidelberg Heidelberg Germany; ^4^ Werner Siemens Imaging Center University of Tuebingen Tuebingen Germany

**Keywords:** arylsulfatase A, metachromatic leukodystrophy, N‐acetylaspartate, neopterin, nuclear magnetic resonance, urine metabolomics

## Abstract

Metachromatic leukodystrophy (MLD) is a lysosomal storage disease caused by a deficiency of the arylsulfatase A (ARSA). ARSA deficiency leads to an accumulation of sulfatides primarily in the nervous system ultimately causing demyelination. With evolving therapeutic options, there is an increasing need for indicators to evaluate disease progression. Here, we report targeted metabolic urine profiling of 56 MLD patients including longitudinal sampling, using ^1^H (proton) nuclear magnetic resonance (NMR) spectroscopy. ^1^H‐NMR urine spectra of 119 MLD samples and 323 healthy controls were analyzed by an in vitro diagnostics research (IVDr) tool, covering up to 50 endogenous and 100 disease‐related metabolites on a 600‐MHz IVDr NMR spectrometer. Quantitative data reports were analyzed regarding age of onset, clinical course, and therapeutic intervention. The NMR data reveal metabolome changes consistent with a multiorgan affection in MLD patients in comparison to controls. In the MLD cohort, N‐acetylaspartate (NAA) excretion in urine is elevated. Early onset MLD forms show a different metabolic profile suggesting a metabolic shift toward ketogenesis in comparison to late onset MLD and controls. In samples of juvenile MLD patients who stabilize clinically after hematopoietic stem cell transplantation (HSCT), the macrophage activation marker neopterin is elevated. We were able to identify different metabolic patterns reflecting variable organ disturbances in MLD, including brain and energy metabolism and inflammatory processes. We suggest NAA in urine as a quantitative biomarker for neurodegeneration. Intriguingly, elevated neopterin after HSCT supports the hypothesis that competent donor macrophages are crucial for favorable outcome.

**Synopsis**

NMR urine profiling in metachromatic leukodystrophy uncovers a multiorgan affection and metabolic switch toward ketogenesis, delineates N‐acetylaspartate as a biomarker for neurodegeneration and identifies neopterin as a possible indicator for disease stabilization after hematopoietic stem cell transplantation.

## 1 INTRODUCTION

Metachromatic leukodystrophy (MLD) is an autosomal recessive lysosomal storage disease caused by a deficiency of the enzyme arylsulfatase A.^1^ Arylsulfatase A deficiency results in the ubiquitous accumulation of sulfatide substrates (3‐O‐sulfogalactosylceramides) in lysosomes. Although MLD is a multisystem disorder affecting kidneys, liver, and gallbladder, the predominant clinical feature is demyelination and subsequently neurodegeneration in the central nervous system (CNS) and peripheral nervous system (PNS).^1^ More rarely an MLD‐like, but genetically and biochemically distinct lipid storage disorder is caused by prosaposin deficiency and saposin B deficiency.^2^ The current diagnosis of MLD is based on clinical and neuroimaging criteria together with biochemical assays and genetic confirmation. Referring to the age of onset, MLD is classified into a late infantile form with age of onset before the age of 2.5 years, a juvenile form with onset between 2.5 and 16 years and an adult form.^3^, ^4^, ^5^ The late infantile form presents mostly with rapid neurodegeneration, whereas the juvenile and adult subtypes exhibit a more variable, but still progressive disease course.^1^, ^4^ To date, the correlation of genotype to biochemical and clinical phenotype is still equivocal and additional parameters such as early clinical symptoms, MRI pattern, ARSA enzyme activity, and sulfatide levels in urine, blood, or cerebrospinal fluid (CSF) are under investigation to predict the disease course.^6^ With advancing therapeutic options like hematopoietic stem cell transplantation (HSCT) and gene therapy, the urge increases to identify easily accessible biomarkers that reflect the disease progression and monitor the treatment course.^7^ Metabolomics has emerged as a potent tool to study biochemical phenotypes of inborn errors of metabolism. Hereby, ^1^H‐NMR spectroscopy at 600 MHz frequency (14.1 Tesla field strength) has proven itself to be a method able to characterize complex biofluids and identify different inborn errors of metabolism reliably.^8^

Here, we report the first quantitative metabolic profiling in urine samples of a large cohort of 56 MLD patients. We analyzed urine metabolite patterns in different subsets of MLD patients and healthy controls by employing an established quantitative IVDr SOP by NMR spectroscopy.^8^ Our in‐depth analysis of metabolites revealed a multiorgan affection and proposed the N‐acetylaspartate (NAA) in urine as a biomarker for neurodegeneration. We further investigate the possibility to discriminate between different forms of MLD based on metabolite clusters. Finally, we provide a metabolic follow‐up of patients with juvenile MLD during HSCT and correlate the clinical course to changes in the urine metabolome to identify the outcome parameters.

## 2 MATERIALS AND METHODS

### 2.1 Study cohort and sample acquisition

One hundred nineteen urine samples of 56 patients with biochemically and/or genetically confirmed MLD have been included into this study comprising 22 late infantile (33 samples), 30 juvenile (80 samples), and 4 adult (6 samples) patients, that is, 22 with early onset (33 samples) and 34 with late onset (86 samples) MLD. Complete demographic data were collected (age range: 0.5–38 years; 34 male samples and 85 female samples). Thirteen patients (44 samples) with juvenile MLD and two patients (seven samples) with late infantile undergoing HSCT as well as five patients (nine samples) with late infantile MLD receiving enzyme replacement therapy (four samples) were identified. Among the juvenile MLD patients, we analyzed data from (10 patients and 16 samples) before HSCT, seven patients (31 samples) who stabilized clinically after HSCT and six patients (13 samples) who showed disease progression after HSCT.^9^ Clinical criteria of disease progression and stabilization were defined according to Beschle and colleagues.^10^ Three hundred twenty‐three urine samples of a healthy control cohort were matched regarding age and gender and used for overall comparison and statistical subsets (age range: 0.06–40 years; 170 males and 153 females). Informed consent was obtained from participating individuals or their legal representatives according to local regulations (ethics number 948/2018BO2).

### 2.2 NMR spectroscopy‐based metabolomics

Defrosted bio‐banked urine samples were thawed up in the fridge and an aliquot of 900 μl urine was taken, mixed with 100 μl of Bruker urine buffer following the Bruker sample preparation standards of procedure (SOPs) as described elsewhere in detail.^8^ ^1^H‐NMR spectra were acquired in full automation with the Bruker's body fluids NMR methods package (B.I.Methods 2.0) using a Bruker Avance IVDr 600 MHz system (Bruker Avance III HD, Ettlingen, Germany) equipped with a 5‐mm triple resonance (TXI) room temperature probe. Bruker's B.I.Quant‐UR1.1 module was used to perform urine metabolite quantification. The limit of detection (LOD) is listed for each metabolite separately (Table S1). MLD samples were collected at the University of Tuebingen, Germany, and the spectra of control samples were provided by Bruker. Methanol can be identified at higher levels in the control samples as a technical artifact due the different processes of sample collection at two different facilities. To avoid any critical effect upon the statistical analysis, methanol was excluded from all univariate and multivariate comparisons.

### 2.3 Statistical analysis

According to the available metadata, full cohort and subgroup statistical investigations were performed with the MetaboAnalyst 5.0 Toolbox.^11^ Corresponding metabolite concentration spreadsheets were normalized with the probabilistic quotient normalization (PQN) method to account for dilution effects. Univariate and multivariate statistical analyses were applied by orthogonal projections to latent structures discriminant analysis (oPLS‐DA), variable importance in projection (VIP) scores, clustered heat maps, volcano analysis, and analysis of variance (ANOVA). Selected metabolites were further investigated with the pattern hunter tool to identify related compounds and mechanism.^12^ Scattered dot and volcano plots were illustrated with GraphPad Prism 9. Detailed statistical data for the complete analyses of all metabolites are listed in Table S1. Metabolites that fall below the limit of quantification (LOQ) are statistically considered as missing values. Variables comprising more than 60% of missing values comparing two cohorts (unpaired *t*‐test) or more than 80% of missing values comparing three cohorts (ANOVA) were excluded from further analyses (Table S1). A false discovery rate (FDR) <0.1 and a raw *p* value <0.05 were applied to account for multiple comparisons as well as facilitating the exploratory detection of biomarkers.^13^, ^14^

## 3 RESULTS

### 3.1 Urine metabolome signature for MLD patients

**FIGURE 1:**
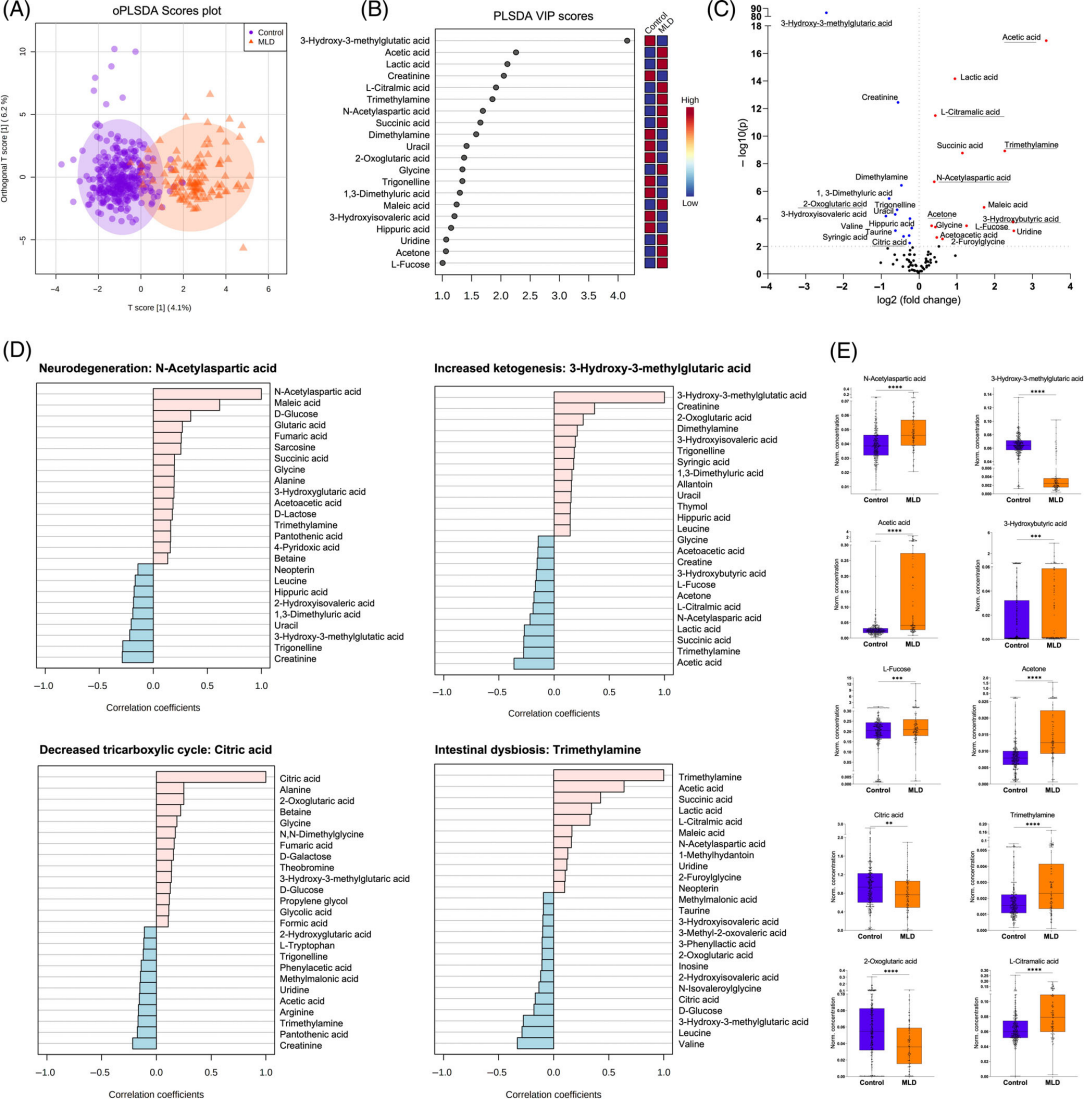
Comparison of urine metabolome in MLD patients versus healthy controls. (A) oPLS‐DA and (B) VIP scores illustrate the relative group overlap analyzing 119 urine samples of 56 MLD patients and 323 urine samples of healthy controls. (C) Volcano plot analysis with upregulated (red) and downregulated (blue) metabolites (FDR < 0.01, *p* < 0.05, fold change > 1.2). Twenty‐seven metabolites are altered significantly. (D) Metabolite pattern investigation and (E) the corresponding dot plots. Whiskers illustrate minimum and maximum. Comparison of metabolites based on unpaired t‐test illustrated in corresponding dot plots, *p* < 0.01 (**), *p* < 0.001 (***), *p* < 0.0001 (****). N‐acetylaspartate (NAA) for brain metabolism, 3‐Hydroxy‐3‐methylglutaric acid (3‐HMG) for ketone body metabolism, citric acid for tricarboxylic cycle and trimethylamine^15^ for gut and urinary tract dysbiosis. FDR, false discovery rate; MLD, metachromatic leukodystrophy; oPLS‐DA, orthogonal projections to latent structures discriminant analysis; VIP, variable importance in projection

NMR metabolomics data of all 119 MLD urine and 323 control samples were analyzed using unsupervised univariant and multivariant regression models to reveal differences between MLD samples and controls. The relative overlap of cohorts is illustrated by oPLS‐DA scores plot and VIP scores (Figure 1A and B). Twenty‐seven metabolites were altered more than 1.2‐fold in the urine collected from MLD patients and healthy controls as depicted in the volcano plot analysis (Figure 1C; Table S1). In detail, an increase in the neurodegenerative marker NAA in MLD urine samples was observed (Figure 1C–E). Moreover, upregulation of ketone bodies (3‐hydroxybutyrate, L‐fucose, acetone, acetic and acetoacetic acid) was detected in samples of MLD patients, whereas 3‐hydroxy‐3‐methylglutaric (3‐HMG) acid decreased in comparison to controls (Figure 1C–E). Tricarboxylic cycle (TCA) metabolites (e.g., citric, fumaric, and 2‐oxoglutaric acid) decreased as well in samples of MLD patients compared to controls (Figure 1C–E). Metabolic indicators for intestinal or urinary tract dysbiosis (trimethylamine [TMA], L‐citramalic acid, and 2‐furoylglycine) were detected at higher levels in the MLD cohort (Figure 1C–E). These metabolic changes regarding ketones, TCA components, gut dysbiosis, and NAA remained persistent comparing solely samples of untreated MLD patients and age‐ and gender‐matched controls (Figure S1A–E).

### 3.2 Metabolic profiling of urine samples comparing early and late onset MLD forms

**FIGURE 2:**
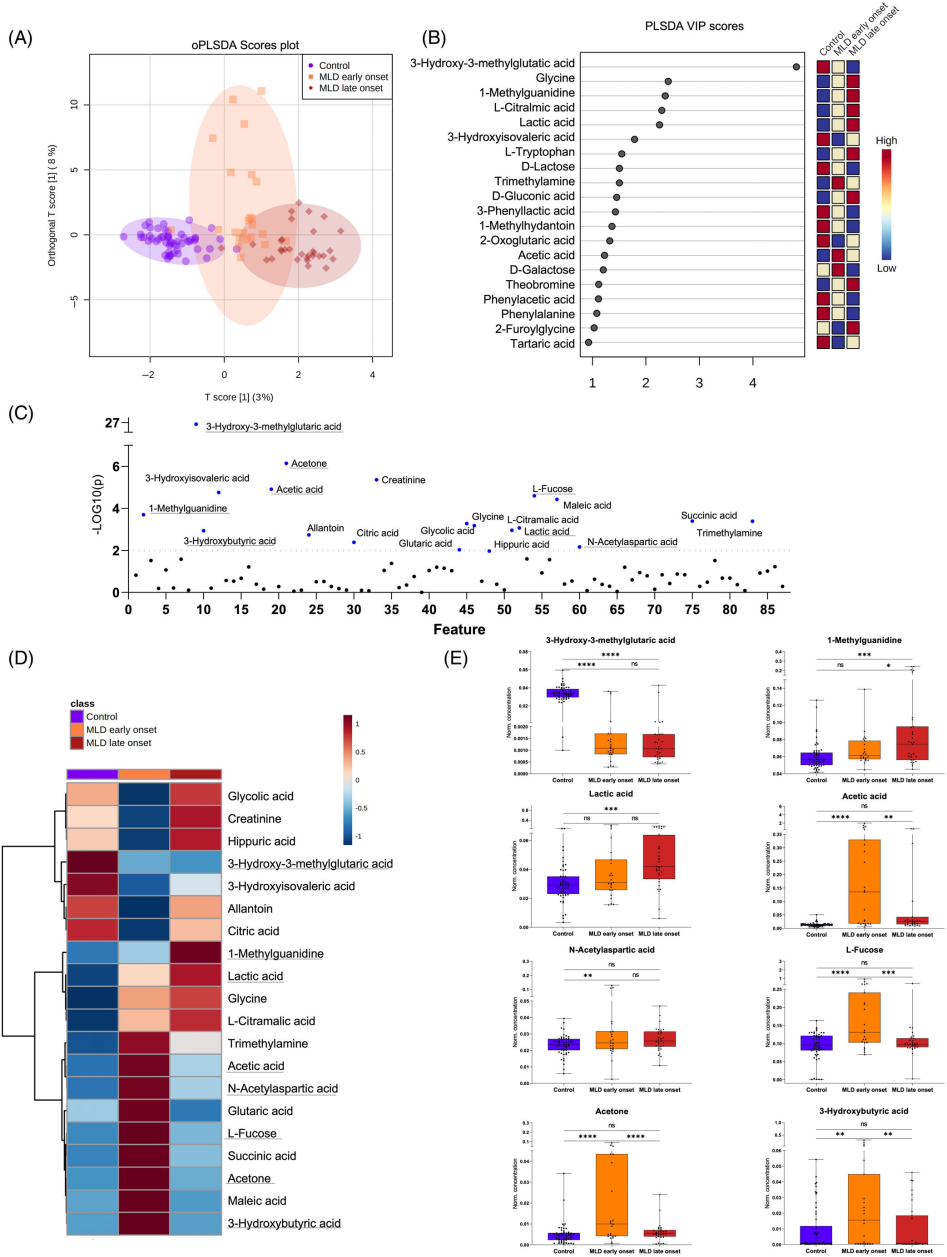
Differentiation between early onset versus late onset MLD in comparison to healthy controls. (A) oPLS‐DA and (B) VIP scores illustrate the relative group overlap analyzing samples of late onset MLD patients (*n* = 31), early onset MLD samples (*n* = 23) and healthy, age‐ and gender‐matched controls (*n* = 54). (C) 20 significant metabolites based on ordinary one‐way ANOVA statistics (*p* < 0.05, FDR < 0.1). (D) Averaged group concentration heat map, suggesting metabolic markers for early onset differentiation (acetic acid, NAA, glutaric acid, L‐fucose, succinic acid, acetone, maleic acid, 3‐hydroxybutyric acid) and late onset (1‐methylguanidine) and their corresponding metabolite concentration dot plots (E). Whiskers illustrate minimum and maximum. Comparison of metabolites based on unpaired t‐test illustrated in corresponding dot plots, *p* < 0.01 (**), *p* < 0.0001 (****). ANOVA, analysis of variance; FDR, false discovery rate; MLD, metachromatic leukodystrophy; oPLS‐DA, orthogonal projections to latent structures discriminant analysis

Next, we analyzed the discriminatory power of urine metabolome profiling to differentiate the cohort of untreated early onset MLD patients (23 samples) from untreated late onset MLD patients (31 samples) and a healthy, age‐ and gender‐matched cohort (54 samples). We illustrated the overlap of cohorts using oPLS‐DA and VIP scores (Figure 2A and B) and identified a total of 20 differing metabolites by ANOVA test (Figure 2C). As depicted in the clustered heat map (Figure 2D), 3‐HMG was downregulated in both MLD subtypes in comparison to controls (Figure 2E). The late onset MLD cohort was characterized by an increase in methylguanidine and lactic acid in comparison to the early onset MLD cohort and controls (Figure 2C–E). The metabolite subset of ketone bodies (acetone, 3‐hydroxybutyric acid, acetic acid, and L‐fucose), maleic and succinic acid as well as NAA were detected at higher levels in the early onset MLD cohort in comparison to samples of late onset MLD patients and healthy controls (Figure 2C–E). In contrast, creatinine, glycolic acid, hippuric acid, allantoin, and citric acid were downregulated in early onset MLD samples.

### 3.3 Identification of prognostic markers for disease progression or disease stabilization after HSCT in juvenile MLD patients

**FIGURE 3:**
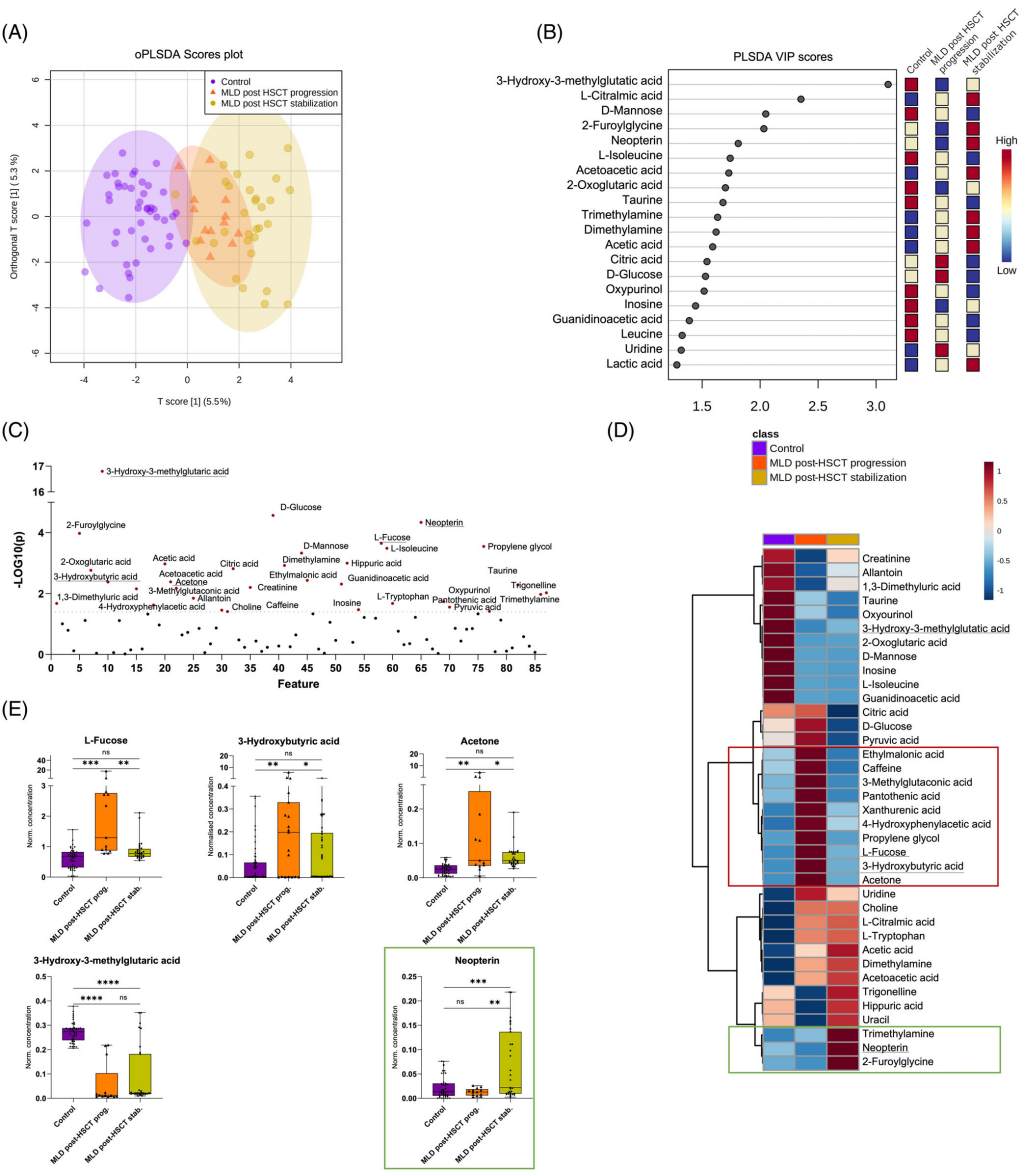
Disease progression versus stabilization after hematopoietic stem cell transplantation (HSCT) in 13 patients with juvenile MLD. (A) oPLS‐DA and (B) VIP scores illustrate the relative group overlap analyzing the cohort with disease progression (13 samples, 6 patients) and stabilization (31 samples, 7 patients) post‐HSCT compared to healthy, age‐ and gender‐matched controls (44 samples). (C) 37 significant metabolites based on ordinary one‐way ANOVA statistics (*p* < 0.05, FDR < 0.1). (D) Averaged group concentration heat map, suggesting metabolite pattern for disease progression (e.g., ketone bodies) or stabilization (trimethylamine, neopterin, and 2‐furoylglycine) after HSCT, and their representative dot plots (E). Whiskers illustrate minimum and maximum. Comparison of metabolites based on unpaired t‐test illustrated in corresponding dot plots, *p* < 0.05 (*), *p* < 0.01 (**), *p* < 0.001 (***), *p* < 0.0001 (****). ANOVA, analysis of variance; FDR, false discovery rate; HSCT, hematopoietic stem cell transplantation; MLD, metachromatic leukodystrophy; oPLS‐DA, orthogonal projections to latent structures discriminant analysis; VIP, variable importance in projection

We conducted follow‐up analyses for 13 patients with juvenile MLD pre‐HSCT and post‐HSCT and correlated the metabolic findings in urine with the clinical disease course. Of these 13 patients, seven stabilized after transplantation clinically, whereas six showed disease progression as defined previously.^10^ NMR metabolite data of these cohorts and healthy controls were analyzed by oPLS‐DA and VIP scores (Figure 3A and B). Thirty‐seven differing metabolites were identified based on ordinary one‐way ANOVA (Figure 3C). The metabolic profile of patients who showed a clinical stabilization after HSCT converged to the control cohort as shown by the averaged group concentration heat map (Figure 3D). The heat map indicated a metabolite cluster for MLD patients with disease progression after HSCT including among other metabolites‐elevated ketone bodies (L‐fucose, 3‐hydroxybutyric acid, acetone) in comparison to patients with disease stabilization and healthy controls (Figure 3D and E). MLD patients with clinical stabilization after HSCT revealed a distinguishing increase in neopterin, TMA and 2‐furoylglycine in comparison to patients with disease progression and controls (Figure 3D and E). In contrast, 3‐HMG remained downregulated in all MLD samples after HSCT (Figure 3D and E). These distinct changes in metabolic profile were not detected before HSCT; however, data were sparse due to a limited number of available samples (Figure S2A–D; Table S1).

### 3.4 Single‐case follow‐ups

**FIGURE 4:**
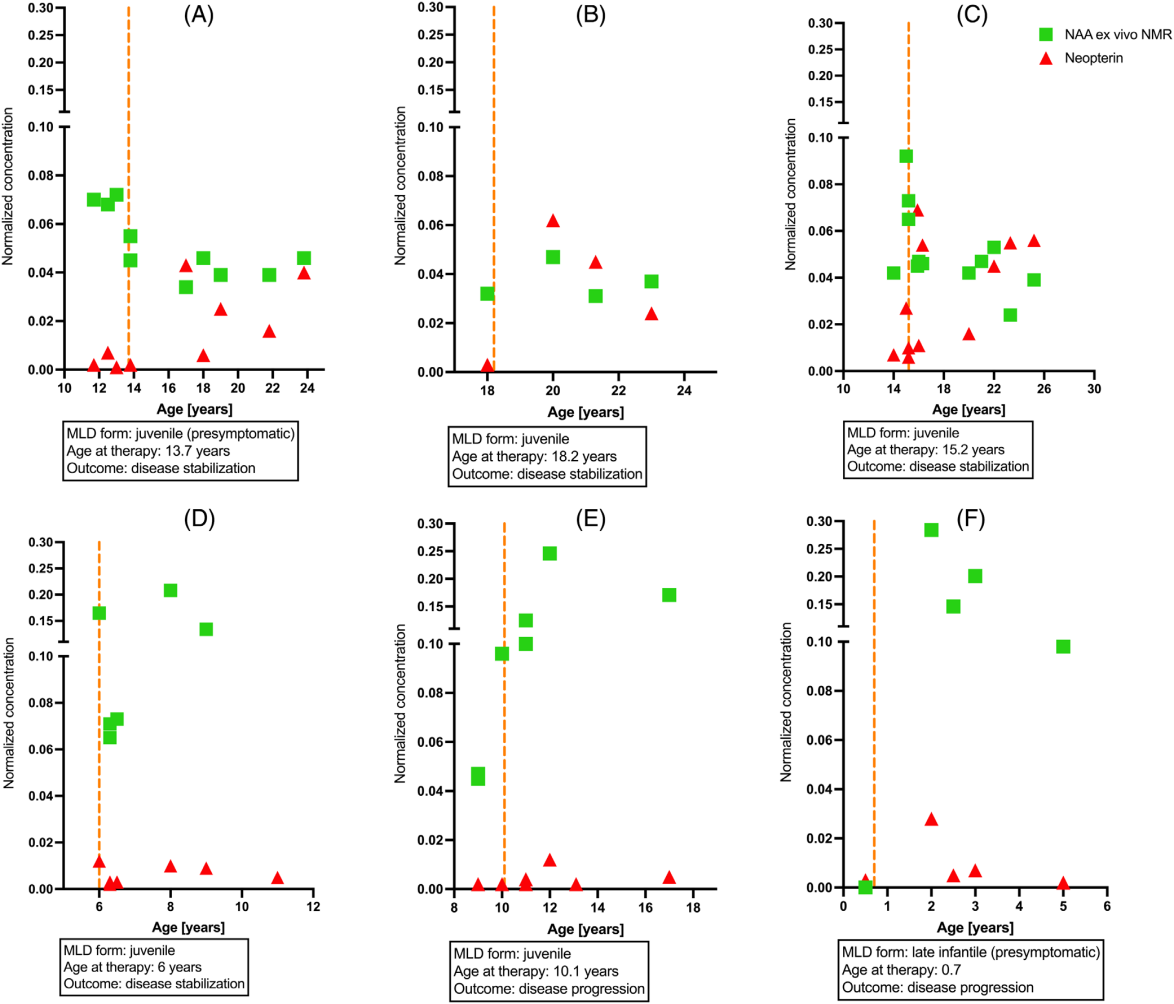
Individual case follow‐up. Representative metabolites were monitored in individual case follow‐up before and after hematopoietic stem cell transplantation (HSCT). N‐acetylaspartate (NAA) (green) and neopterin (red) measured ex vivo by NMR in urine. Time of hematopoietic stem cell transplantation (HSCT) (orange line). NMR, nuclear magnetic resonance

To evaluate the urinary metabolome profiles for individual disease monitoring and follow‐up, we investigated several affected individuals in a long‐term follow‐up before and after HSCT (Figure 4). We identified three individuals with juvenile MLD, who showed long‐term stabilization after HSCT without or with only minimal functional impairment (Figure 4A–C). All of them reveal an increase in neopterin after HSCT and decreasing NAA levels in urine. Individual D exhibited an initial disease stabilization, but with a significant loss of cognitive and motor functions in a long‐term follow‐up. In this follow‐up, NMR profiling detected elevated NAA levels after HSCT and no increase in neopterin. Individual E with juvenile MLD and individual F with late infantile MLD clinically exhibited a progression of the disease, which was accompanied by considerably elevated NAA levels, whereas an increase in neopterin was minimal and not permanent.

## 4 DISCUSSION

The application of quantitative NMR spectroscopy‐based urine metabolomics was evaluated for the first time in a large cohort of MLD patients. The data suggest that NMR‐based IVDr SOPs for metabolomics is a feasible tool to investigate urine profiles in MLD patients compared to healthy controls. The oPLS‐DA reveals a separation between the MLD cohort and controls (Figure 1A; Figure S1A) and further in‐depth analyses suggest several metabolites as pathophysiological indications for disease severity and progression under treatment (Figure 1D and E; Figure S1D and E).

### 4.1 Multisystemic readout of urine metabolome in MLD patients reveals multiorgan affection

Urine is a biofluid that reflects the body's struggle to regulate metabolic and osmotic homeostasis. When investigating different metabolite correlation patterns, we identified several metabolic variables that indicate a multiorgan affection in MLD (Figure 1D) according to the Human Metabolome Database.^16^, ^17^, ^18^

#### 4.1.1 Brain metabolism

We detected an increase in NAA as a specific neuronal biomarker for neurodegeneration in urine samples of MLD patients. NAA is synthesized from aspartate and acetyl coenzyme A (acetyl CoA) in neurons. Among various other functions, it serves as a source of acetate for lipid and myelin synthesis in oligodendrocytes and is a precursor of the neurotransmitter N‐acetylaspartylglutamate. Notably, we could exclude nutritional supplementation of NAA based on the metadata available. NAA has been identified as a endogenous marker of neuronal injury and acts in high concentration as neurotoxic and induces oxidative stress.^19^ Chronically high levels of NAA in urine have been also detected in Canavan disease.^20^ Interestingly, NAA increase was pronounced in early onset MLD patients who exhibited the most progressive neurodegenerative disease course. Of note, an up to 6‐fold higher NAA and 10‐fold higher maleic acid concentrations were detected in three samples of one patient with the late‐infantile onset and rapidly progressing neurodegeneration (Figures 1E and 2E).

This finding correlates well with data from cerebral proton MR spectroscopy (MRS) that revealed a decrease of NAA with progressive neurodegeneration,^21^, ^22^ also confirmed in our patients (data not shown). The accumulation of sulfatides initially leads to demyelination that is detectable in white matter alterations. Over the disease course MRS data of MLD patients reveal a reduction of NAA in gray and white matter as a sign of neuronal and axonal loss.^22^ van Rappard and colleagues showed that NAA levels measured by MRS could predict the outcome after HSCT for juvenile forms of MLD.^22^ In late infantile MLD cases, the decrease in NAA levels in MRS is reported to correlate with the decline of neurological function.^21^

#### 4.1.2 Ketone body metabolism

Besides the tissue‐specific cerebral metabolite NAA, we identified glycolysis/TCA and ketone body metabolism pathways as of high impact to distinguish between the MLD cohort and controls. The upregulation of ketones (3‐hydroxybutyrate, L‐fucose, acetone, acetic, and acetoacetic acid) along with a decrease of 3‐HMG (Figure 1D and E) argues for an increased ß‐oxidation.^23^ The brain metabolism requires 20% of available glucose.^24^ Besides that, the brain can metabolize ketones as an alternative energy source. Under stress due to pathological processes such as MLD, brain glucose consumption can become massive and finally the metabolism shifts toward ketone body formation in the liver and to a minor extent astrocytes and kidneys.^23^, ^25^, ^26^ The decrease of TCA components is also consistent with a ketogenic switch (Figure 1D and E). A comparable shift in cerebral energy metabolism concomitant with demyelination and neurodegeneration has previously been suggested after analysis of the urine metabolome of multiple sclerosis patients.^27^ Increased maleic, fumaric, and glutaric acid might also suggest a demanding cellular and mitochondrial energy metabolism. Mitochondrial affection and/or dysfunction has been discussed in other lysosomal storage disorders,^28^, ^29^ but for MLD a precise pathomechanism has not yet been established. Moreover, L‐fucose, acetone and 3‐hydroxybutyrate secretion in the urine might as well result from liver damage.^30^, ^31^ An impairment in liver function is supposed to occur in MLD due to the accumulation of sulfatides in hepatocytes.^32^ Consequently, metabolites associated with hepatic energy metabolism and oxidative stress might reflect both, an increased cerebral energy demand secondary to neurodegenerative processes as well as an affection of hepatic cell metabolism.

Interestingly, the elevation of liver‐associated metabolites was predominant in samples of late‐infantile MLD patients (Figure 2D) and juvenile MLD patients, who showed disease progression after HSCT (Figure 3D). We analyzed outliers with an up to 10‐fold increase in L‐fucose, acetone, and 3‐hydroxyburyrate and identified several samples of two patients with far advanced late‐infantile MLD and a sample of one patient with juvenile MLD onset and moderate disease progression, but significant gallbladder affection (Figures 1E and 2E).

#### 4.1.3 Gut metabolism

In comparison to controls, several markers of intestinal and/or urinary tract dysbiosis are elevated in MLD urine samples (Figure 1D and E). TMA is a microbial metabolite and its presence in urine indicates an intestinal dysbiosis as TMA is excreted by various gut bacteria.^33^, ^34^, ^35^ However, the average increase in TMA in the cohort of juvenile MLD patients that stabilize after HSCT might result from the statistical variance due to few outliers and more data are needed to evaluate this observation. The elevation of 2‐furoylglycine has been associated with changes in gut microbiome and nutrition as well as an altered mitochondrial fatty acid ß‐oxidation^36^, ^37^ (Figure 3D and E). Increased levels have been reported in the early stages of Parkinson's disease.^36^ However, further functional studies are needed to evaluate a possible pathophysiological role in MLD before and after HSCT (Figure 3D and E).

L‐citramalic acid is another marker not formed in human tissues but synthesized by anaerobic bacteria or yeast and hence its elevation in MLD samples might indicate urinary and/or intestinal dysbiosis (Figure 1D and E).^38^

#### 4.1.4 Inflammation

In other human studies on inflammatory disorders, several metabolite clusters were identified as indicators for inflammatory changes including lactic acid, succinic acid, and acetic acid^39^, ^40^ (Figure 1B and C). Interestingly, in multiple sclerosis patients, the urine metabolome revealed a similar pattern.^27^ In MLD, inflammation has been discussed and studied as a contributing factor of disease progression over time.^41^, ^42^ It has been shown that sulfatides induce a significant inflammatory response and stimulate microglia.^43^ Especially, in samples of patients with juvenile disease onset at advanced ages (>20 years) or of patients with early onset in acute decline, the increase in lactic and acetic acid was predominant (Figure 2E).

Nonetheless, these metabolites are involved in various pathways and can be also produced by microbiota and thus further functional studies are needed to elucidate whether and to what extent these metabolic findings are associated with an MLD‐specific pathophysiology.

#### 4.1.5 Different metabolite patterns in early and late MLD forms

Our results suggest metabolic differences between MLD subtypes based on metabolite patterns in urine. The variables contributing to the separation of early onset MLD from late onset and controls included metabolites that reflect altered liver and ketone body metabolism (e.g., 3‐hydroxybutyric acid, L‐fucose, acetone, and acetic acid), likewise inflammation (lactic and acetic acid), and neurodegeneration (NAA). This metabolic signature most likely reveals the degree of multisystem affection in early onset MLD forms correlating with the rapidly progressive disease course. Interestingly, a comparable pattern regarding energy, liver, and brain metabolism is detected in samples of patients with juvenile MLD, who suffer from disease progression after HSCT (Figures 3D and 4).

The late onset MLD cohort differs from healthy controls and early onset MLD predominantly by an increase in methylguanidine. Notably, methylguanidine has a strong anti‐inflammatory effect^44^; however, further studies are required to investigate a possible pathophysiological impact in late onset MLD.

The analysis of different MLD subtypes indicated that a subset of metabolites has some potential to differentiate between these cohorts in symptomatic patients. However, these metabolic clusters are not associated with a distinct pathophysiological mechanism and rather mirror the extent of multisystem involvement. To analyze the predictive value of NMR urine metabolome data to project the MLD subtype and disease course of yet asymptomatic MLD patients, further studies are warranted.

#### 4.1.6 Neopterin as a biomarker for a therapeutic response after HSCT

Over the last decades a number of MLD patients have been treated with hematopoietic stem cell transplantation,^10^, ^32^, ^45^ which showed that the transplantation of patients with late‐infantile MLD is not beneficial as the disease progresses too rapidly.^46^, ^47^ Late onset forms, however, might benefit when transplanted in the early stages of the disease. Although various predicting factors have been identified clinically,^10^ biochemical parameters to project the clinical outcome are still lacking. We detected a significant increase in neopterin exclusively in juvenile MLD patients who stabilized after HSCT (Figure 3D and E). Neopterin is involved in pterin biosynthesis and serves as a biomarker for increased phagocytic function in macrophages and microglia.^48^ Phagocytes including microglia in the nervous system require the ARSA enzyme to decay sulfatides in their lysosomes. ARSA deficient phagocytes, however, accumulate lysosomal vesicles and eventually induce apoptosis.^15^ According to previous studies, phagocyte death and lysosomal breakdown precede the myelin and consequently neurodegeneration in MLD.^15^ Hence, massive cell damage or death of microglia might contribute substantially to the neurodegenerative process seen in MLD.^15^, ^49^ After HSCT, however, activated donor macrophages were detected throughout the white matter and were supposed to play a major protective role for the remaining oligodendrocytes and neurons.^50^ A comparable neuroprotective effect due to functional microglia after HSCT has been described in Sandhoff disease, another lysosomal storage disorder.^51^ Here, we show that neopterin increases in patients who show clinical stabilization after HSCT (Figure 3E). The elevated neopterin validates the pathophysiological candidate mechanism that competent phagocytic activity is crucial to prevent disease progression after HSCT. Although Wolf and colleagues^50^ showed that monocyte‐derived donor macrophages were present widespread in the white matter after HSCT and hypothesized their immunological key role for neuroprotection and remyelination, their study included mostly patients with disease progression after transplantation. In contrast, our findings show that neopterin increases only in patients with disease stabilization. As neopterin is not detected in these cohorts before transplantation (Figure S1A–D), the crucial contribution of donor‐phagocytes is emphasized. Neopterin in urine has been used as a reliable biomarker of graft versus host reaction after organ transplantation and/or as an indicator of viral infection.^52^ Recently, it was shown that neopterin is markedly elevated in the active phase of COVID‐19 infections.^53^ However, we could exclude underlying graft versus host disease or viral infections by studying the individual patients with elevated neopterin levels in urine in all patients.

### 4.2 Limitations

The major limitation of this study results from relatively small cohorts (and subgroups) for statistical metabolome analysis. Due to multiple exogeneous factors urine metabolome analyses underly considerable undulations and this might explain in part the statistical variation. Most importantly, we would like to emphasize that any hypothesis on brain metabolism formulated based on data from urine samples studies faces obvious constraints. We would favor the most stringent statistical analyses together with a rigorous classification of clinical subgroups; however, the rareness of disorder and hence sample availability imposes an innate limitation to this study. Accounting for the fact that we seek to discover biomarkers and generate hypotheses an FDR < 0.1 was allowed. Future studies in larger cohorts and from different human tissues and body fluids are certainly required to improve the statistical power and thereby evaluate potential biomarkers identified in this study. We did not analyze presymptomatic newborns to evaluate the predictive value of NMR metabolome profiling. Indeed, such analysis will be crucial in the development of newborn screening for MLD.^54^ Furthermore, we lack comparable studies in other lysosomal storage disorders and, in general, in pediatric multisystem disorders. The metabolic pattern identified reflect the degree of ubiquitous organ affection and hence secondary effects. Other disorders might display comparable profiles with disease progression, but there are no data of statistically meaningful cohorts yet available.

Nevertheless, it is a considerable advantage that using a highly standardized NMR spectroscopy‐based protocol facilitates full comparability with future NMR studies and hereby overcomes an intrinsic challenge of rare diseases: the limited number of available patients and samples.

## 5 CONCLUSION

This study provides the first global urine metabolic profiling by NMR spectroscopy‐based metabolomics in an MLD cohort. Different metabolome panels revealed a multiorgan affection and metabolic switch towards ketogenesis in MLD patients. Early onset and late onset MLD patients reveal differing metabolite clusters. We identified NAA in urine as a biomarker for neurodegeneration. A significant increase in neopterin in the urine of juvenile MLD patients indicated a disease stabilization after HSCT in a longitudinal follow‐up. Metabolome profiling by NMR spectroscopy is a promising tool for routine diagnostics to elucidate disease mechanisms and monitor neurodegenerative disorders. However, the limited sample size argues for considering the identified metabolome signatures only as a hypothesis that has the potential to develop into definite metabolic characterization for MLD disease monitoring over time.

## CONFLICT OF INTERESTS

Judith Böhringer archived biomaterial. Samuel Groeschel and Ingeborg Krägeloh‐Mann are members of the European Reference Network for Rare Neurological Diseases, project ID 739510. Samuel Groeschel received institutional research support from Shire plc. He is an advisor and a coinvestigator for trials in MLD (Shire/Takeda, Orchard, and Bioclinica) but receives no personal payment related to this role. Ingeborg Krägeloh‐Mann received travel funds from Shire/Takeda. Lucia Laugwitz, Laimdota Zizmare, Vidiyaah Santhanakumaran, Claire Cannet, Judith Böhringer, Jürgen G. Okun, Manfred Spraul, and Christoph Trautwein declare that they have no conflict of interests.

## ETHICS STATEMENT

The author(s) confirm(s) independence from the sponsors; the content of the article has not been influenced by the sponsors. Details of ethics approval A patient consent statement was obtained according to local standards (ethics number 948/2018BO2).

## Supporting information


**Figure S1**: Comparison of urine metabolome in untreated MLD patients (*n* = 54) versus healthy, age‐ and gender‐matched controls (*n* = 54). (A) oPLS‐DA and VIP scores illustrate the relative group overlap. (B) Volcano plot analysis with upregulated (red) and downregulated (blue) metabolites (FDR < 0.01, *p* < 0.05, fold change > 1.2). Eighteen metabolites are altered significantly. (C) Metabolite pattern investigation with the corresponding dot plots. Whiskers illustrate minimum and maximum. Comparison of metabolites based on unpaired t‐test illustrated in corresponding dot plots, *p* < 0.0001 (****). N‐acetylaspartate (NAA) for brain metabolism, 3‐Hydroxy‐3‐methylglutaric acid (3‐HMG) for ketone body metabolism, citric acid for tricarboxylic cycle and trimethylamine (Bergner et al) for gut and urinary tract dysbiosis.

**Figure S2:** Disease progression versus stabilization before hematopoietic stem cell transplantation (pre‐HSCT) in patients with juvenile MLD. (A) oPLS‐DA and (B) VIP scores illustrate the relative group overlap analyzing the cohort with disease progression (four patients and six samples) and stabilization (6 patients and 10 samples) in juvenile MLD patients before HSCT compared to age‐ and gender‐matched controls (16 samples). (C) Six significant metabolites based on ordinary one‐way ANOVA statistics (*p* < 0.05, FDR < 0.1). (D) Metabolite pattern investigation with the corresponding dot plots (E). Whiskers illustrate minimum and maximum. Comparison of metabolites based on unpaired t‐test illustrated in corresponding dot plots, *p* < 0.0001 (****).

**Table S1:** Statistical analyses including minimal (min), maximal (max) values, 95% confidence interval, standard deviation (StDev), median and *p*‐values.

## Data Availability

Additional data are available upon request.
