# Supplementary figures and images for: Identification of neurodegeneration indicators and disease progression in metachromatic leukodystrophy using quantitative NMR‐based urinary metabolomics
Source: JIMD Rep. 2022 Jan 27;63(2):168–80. doi: 10.1002/jmd2.12273 (PMC8898726; doi:10.1002/jmd2.12273)

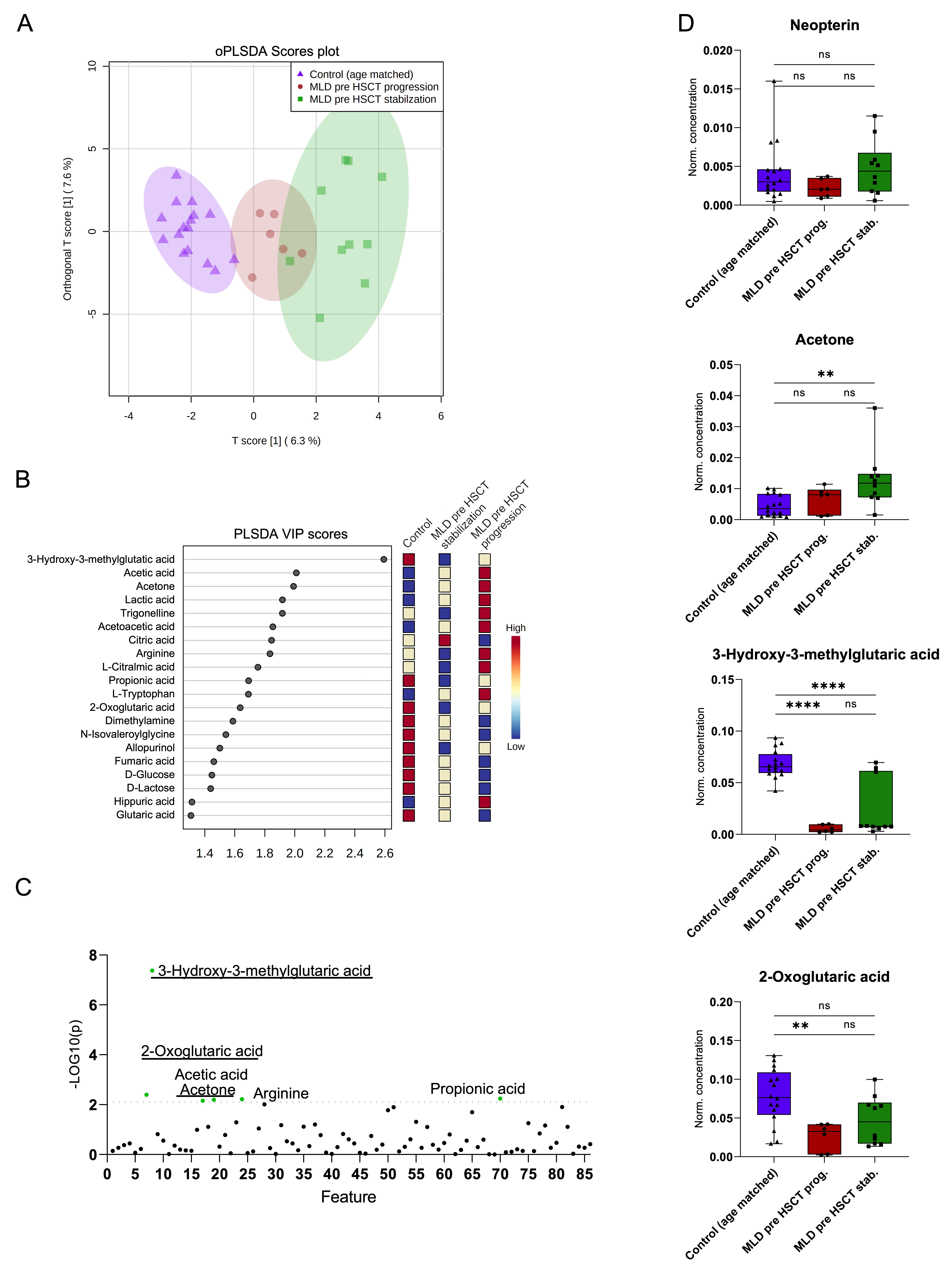

Supplement: Supplementary file 2 — Figure S2: Disease progression versus stabilization before hematopoietic stem cell transplantation (pre‐HSCT) in patients with juvenile MLD. (A) oPLS‐DA and (B) VIP scores illustrate the relative group overlap analyzing the cohort with disease progression (four patients and six samples) and stabilization (6 patients and 10 samples) in juvenile MLD patients before HSCT compared to age‐ and gender‐matched controls (16 samples). (C) Six significant metabolites based on ordinary one‐way ANOVA statistics (p < 0.05, FDR < 0.1). (D) Metabolite pattern investigation with the corresponding dot plots (E). Whiskers illustrate minimum and maximum. Comparison of metabolites based on unpaired t‐test illustrated in corresponding dot plots, p < 0.0001 (****). [file JMD2-63-168-s002.jpg]
